# Behavioural mimicry as an indicator of affiliation

**DOI:** 10.1371/journal.pone.0250105

**Published:** 2021-05-03

**Authors:** Héctor M. Manrique, Antonio Marín, Paula Andrea Nieto-Alemán, Dwight W. Read, Janeth Hernández-Jaramillo, Azucena García-Palacios, Henriette Zeidler

**Affiliations:** 1 Department of Psychology and Sociology, Universidad de Zaragoza, Zaragoza, Spain; 2 Department of Basic and Clinical Psychology and Psychobiology, Universitat Jaume I, Castellón, Spain; 3 Valencian International University, Valencia, Spain; 4 Business & Marketing School ESIC, Valencia, Spain; 5 Department of Anthropology and Department of Statistics, University of California, Los Angeles, California, United States of America; 6 Escuela de Medicina y Ciencias de la Salud, Universidad del Rosario, Bogotá, Colombia; 7 Institute of Health Carlos III, Madrid, Spain; 8 Aston University, Birmingham, United Kingdom; 9 University of Gothenburg, Gothenburg, Sweden; University of Minnesota, UNITED STATES

## Abstract

Previous research has shown that behavioural mimicry fosters affiliation, and can be used to infer whether people belong to the same social unit. However, we still know very little about the generalizability of these findings and the individual factors involved. The present study intends to disentangle two important variables and assess their importance for affiliation: the matching in time of the behaviours versus their matching in form. In order to address this issue, we presented participants with short videos in which two actors displayed a set of small movements (e.g. crossing their legs, folding their arms, tapping their fingers) arranged to be either contingent in time or in form. A dark filter was used to eliminate ostensive group marks, such us phenotype or clothing. Participants attributed the highest degree of affiliation to the actors when their subsequent movements matched in form, but were delayed by 4–5 seconds, and the lowest degree when the timing of their movements matched, but they differed in form. To assess the generalizability of our findings, we took our study outside the usual Western context and tested a matching sample of participants from a traditional small-scale society in Kenya. In all, our results suggest that movements are used to judge the degree of affiliation between two individuals in both large- and small-scale societies. While moving in different ways at the same time seems to increase the perceived distance between two individuals, movements which match in form seem to invoke closeness.

## Introduction

Mimicry is usually defined as the intentional or unintentional reproduction of a model’s postures and mannerisms, facial expressions, emotions, and accents [1–3]. The factors that trigger interpersonal mimicry have been extensively investigated. It is known, for instance, that things as trivial as sharing first names [4], or wearing the same scarf or armband [5] can elicit mimicking behaviours. Internal factors such as liking or wanting to affiliate with someone have been shown to increase mimicry as well [6–8]. The effects of being mimicked can be summarized as inducing a more positive attitude towards the mimicker [9,10], such as facilitating acceptance [8,11] or creating the feeling that the mimicker is more trustworthy [12]- all of which increase affiliation. Fisher and Hess [13] even showed that people mimic others’ emotional expressions only when there is a potential for affiliation. Mimicry thus seems to serve as “social glue” which demonstrates similarity to others [14]. However, the affiliative effect seems to depend on a tight set of parameters. If the mimicking of the interaction partner does not occur within the right temporal window (2 to 5 seconds later), is too exact, or too obvious, the imitation can even backfire and produce negative results such as rejection [15,16].

A different phenomenon which has been shown to generate affiliation is interpersonal synchrony or *entrainment*. Unlike mimicry, which does not require external cues, entrainment refers to people aligning their movements by following an external rhythm or tempo (see [17] for an introduction). Lakens and Stel [18] investigated interpersonal synchrony and found that participants watching a video of two people waving at the exact same rhythm attributed high levels of entitativity and rapport (i.e. affiliation) to the wavers. Interestingly, when participants were asked to attribute entitativity and rapport to two walking individuals who were thought to have been instructed to synchronize their gait, the levels dropped. Interpersonal synchrony thus seems to evoke entitativity and rapport mainly when it is felt to happen spontaneously. Remarkably, the same effect can already be found in young infants. Cirelli, Einarson, & Trainor [19] showed that fourteen-month-old infants were more helpful towards an adult who had bounced in synchrony with the adult bouncing the infant than to one who had bounced to a different rhythm. In the same vein, a study by Fawcet and Tuncgenc [20] found that fifteen-month-olds inferred that two characters who had moved in synchrony would affiliate with each other. Further research has shown that even auditory information is enough to evoke affiliative effects and that hearing two persons walking together involves a wider brain network than hearing footsteps from a single person [21]. Miles, Nind, & Macrae [22] found that the mode of interpersonal synchrony strongly affects affiliation, with the most stable forms of coordination (in-phase and anti-phase movements) leading to the highest levels of rapport. Catmur and Heyes [23] investigated the influence of similarity and contingency on prosocial behaviours and affiliation, and found that an increase in both outcomes was linked to the temporal contingency between participants’ actions and those of their interaction partners, regardless of whether the partners’ actions were the same as their own. Similarly, Hove and Risen [17] found that having two people tapping fingers in synchrony induced feelings of liking and the effect was due to the timing of the movements rather than its matching in form.

These latest two studies showed the importance of disentangling the individual factors involved in interactive behaviours, and led us to assume that we would find the strongest effect of affiliation when actions happened at the same time, followed by their similarity in form. The aim of the current study was thus to further explore the individual effects of these variables and assess whether it is temporal contingency of movements or their contingency in form which is decisive in judging whether the interacting individuals form a social unit. Since our question did not focus on entrainment, we completely uncoupled contingency in time from contingency in form and thus created a total of three conditions: temporal contingency (actors move at the same time, but in a different way), form contingency (actors move at different times, but in the same way), and a control condition in which actors moved at different times in different ways. Another important aspect which differentiates our study from previous work is the inclusion of a non-WEIRD sample to test the generalizability of our results.

## Study I

### Method

#### Participants

A total of sixty-five university students from Spain aged between 18 and 37 years took part in the experiment. Forty-nine students (41 female, 8 male) came from the University of Zaragoza (M_*age*_ = 19.14, SD = 1.63) and sixteen students (15 female, 1 male) came from the University Jaume I (M_*age*_ = 25.5, SD = 3.75). Participation was fully voluntary and not part of the students’ course work. The study was approved by the ethical committee of University Jaume I and adhered to all applicable standards. Informed written consent was obtained from the participants prior to commencing the study.

#### Materials

**Fig 1:**
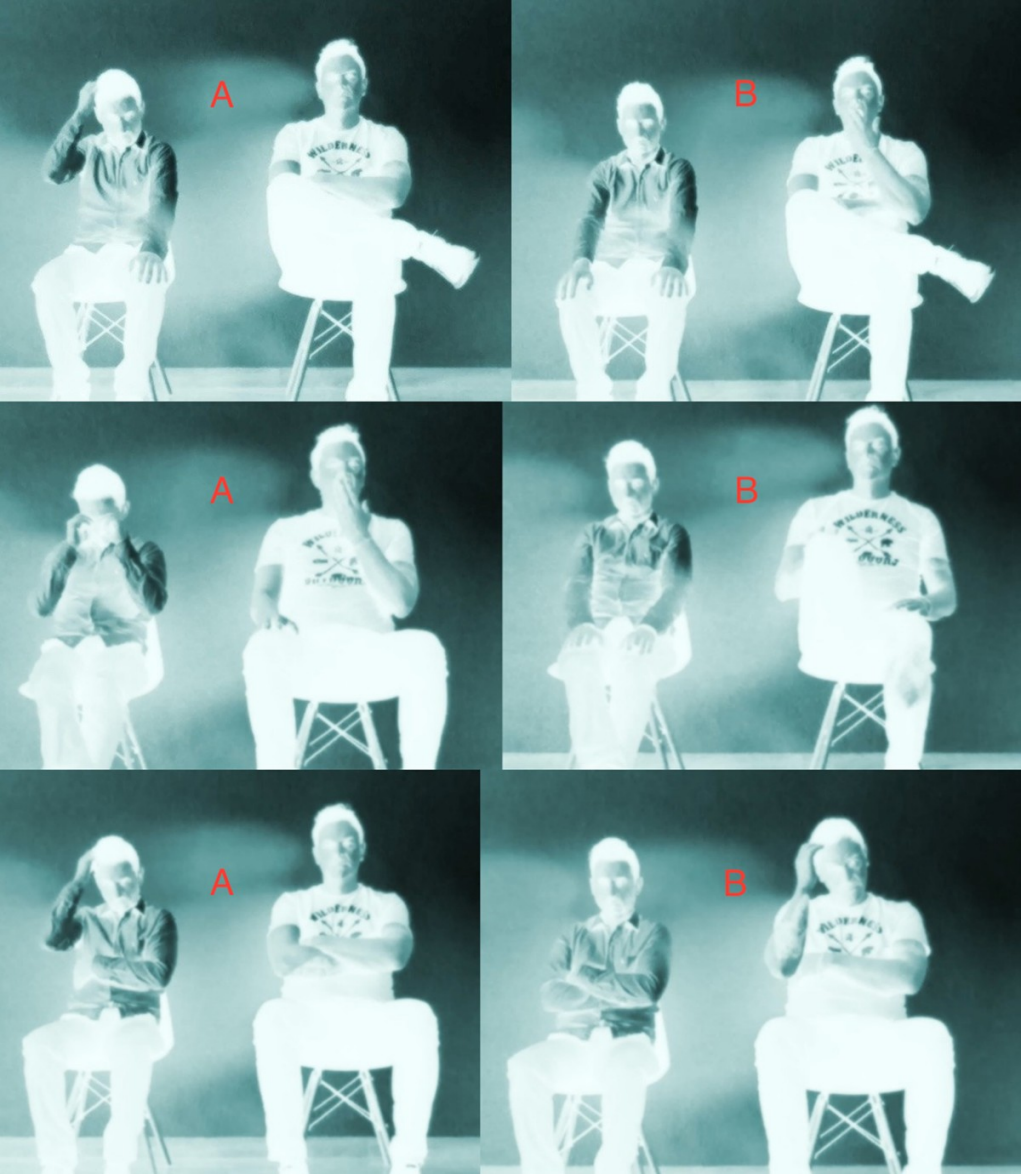
Screenshots of typical sequences (A-B) from the three conditions (dark filter largely removed for better image clarity—see SOM for original videos). Top: No Contingency condition. Sitting in different positions, actor 1 scratches his head while actor 2 remains still (A). Eleven seconds later actor 2 starts to yawn, while actor 1 retains his previous position (B). Middle: Temporal Contingency condition. Sitting in different positions, actor 1 gently rubs his eyes while actor 2 yawns simultaneously (A). Twenty seconds later, actor 2 starts crossing his legs, and actor 1 simultaneously starts tapping his fingers on his knees (B). Bottom: Form Contingency condition. Sharing the same overall position, actor 1 scratches his head with his right hand while actor 2 remains still (A). Four seconds later, actor 2 scratches his head with his right hand while actor 1 remains still (B).

We recorded three different videos in which two actors were seated side by side in two chairs which were 0.5 meters apart and whose backseats touched the rear wall. During recording, the actors always looked to the front. Because participants were to rate the degree of affiliation between the actors, we avoided a face to face arrangement. Having the actors sitting face to face for five minutes without talking would create a very artificial situation, and having them talking would create the impression they already knew each other. Recording them side by side created a more natural situation which could apply to strangers in a waiting room, but also to relatives or friends who know each other so well that they have nothing new to contribute for 5 minutes. Across all conditions, both actors displayed a series of small gestures or body movements, including stretching or crossing their legs, folding their arms, tapping their fingers, rubbing their eyes, or yawning. A detailed description can be found in the Supporting Online Materials. Both actors showed the same set of behaviours in each of the three videos, but their contingency was manipulated in order to create the following conditions: 1) No Contingency condition: Postures and movements were arranged to avoid any contingency between the actors. Breaks between individual actions were always longer than 8 seconds, and the actors’ subsequent movements never matched in form. 2) Temporal Contingency condition: Both actors moved at exactly the same time, but their postures never matched in form; and 3) Form Contingency condition: Every time an actor showed a certain behaviour or adopted a new posture, the other did the same 4 to 5 seconds later, so that there was a perfect, albeit slightly delayed, match between both actors. To make the recorded situation appear more natural, the actors took turns in changing postures. Because the goal of the study was to see whether movement alone can help to ascertain affiliation, we used a potent video filter to obscure ostensive potential group markers such us phenotype or style of clothing. Therefore, what was seen in the videos consisted mainly of the silhouettes of both actors and their movements. Fig 1 provides screenshots of the three experimental conditions with a most of the dark filter removed for better clarity. In the actual test, the dark filter was much stronger so that only the silhouettes of the actors remained visible. The duration of all three videos was 5 minutes and 15 seconds, with a total of 10–15 changes in position per actor.

#### Design and procedure

Participants were randomly assigned to one of the three conditions: No Contingency (N = 22, M_*age*_ = 19.5, SD = 1.89), Temporal Contingency (N = 21, M_*age*_ = 21.8, SD = 4.05), and Form Contingency (N = 22, M_*age*_ = 21, SD = 4.33). They were instructed to watch the video they had been assigned to with great attention because later they would have to answer a question relating to what they had just watched. When the video was over, they were asked to rate the degree of affiliation between the two actors on a scale from a minimum of 1 to a maximum of 7. To help them judge, subjects were told that a rating of 1 could be equated to two people from different countries who had never met before; a rating of 4 (the intermediate value) would be equivalent to two people who shared their nationality, yet were unacquainted; and a rating of 7 (the maximum value on our scale) would be equivalent to father and son or two brothers. We emphasized that these labels were only to illustrate the degree of affiliation between the actors, and did not imply the actual relationship between them. Subjects were also reminded that they could use any number on our scale from 1 to 7 to illustrate the perceived degree of affiliation between the actors.

#### Statistical procedures

The data were analyzed using two-way ANOVA with the post hoc Tukey HSD (Honest Significant Difference) test (XLSTAT version 2021.1) for determining which ranking contrasts have significantly different means at the Group (Samburu or Spanish) and Contingency (No, Temporal, or Form Contingency) levels.

Prior to the two-way ANOVA, we did a preliminary statistical analysis to determine if any modifications in the data set were needed before doing a two-way ANOVA analysis of the rankings by Group and Contingency. We found a single data case (Group = Samburu, Contingency = Form, and Ranking = 1) with a standardized z-score (z = -3.78, p = 0.00008) that justifies its removal from the data set as an outlier. Next, we tested for an interaction between Group and Contingency and found that the null hypothesis, H_0_: No Group * Contingency Interaction, is accepted at the α = 0.30 significance level (df = (2, 119), F = 0.927, p = 0.399). Next, we found that the null hypothesis, H_0_: Data are Homoscedastic, is accepted at the α = 0.05 level for the Group variable (df = (1,120), F = 3.51, p = 0.064) and accepted at the α = 0.70 level for the Contingency variable (df = (2, 120), F = 0.25, p = 0.78). Lastly, the null hypothesis, H_0_: Data Are Normally Distributed, can be rejected at the α = 0.001 level using the Shapiro-Wilks test (W = 0.97, p = 0.008). However, since the two-way ANOVA is robust with respect to moderate deviations from normality and all the data are unimodally distributed, no correction for non-normality was considered to be needed.

### Results

The results for the three contingency conditions are shown in Fig 1 (lower graph in red). Visually, the Form Contingency condition has the greatest effect on the affiliation rankings, followed by the No Contingency and then the Temporal Contingency condition.

In contrast with previous research, we found a statistically significant difference at the α = 0.05 level for the No Contingency compared to the Form Contingency conditions (Tukey’s standardized d score = 2.47, p = 0.014). Surprisingly, there was no significant difference at the α = 0.90 level between the No Contingency and the Temporal Contingency conditions (Tukey’s standardized d score = 0.859, p = 0.96). The failure to find a significant difference is not likely to result from low power for the test due to small sample size since the difference was not significant even at the α = 0.90 level. The affiliation scores in the Temporal Contingency condition were significantly different at the α = 0.001 level in comparison to the Form Contingency condition (Tukey’s standardized d score = 3.30, p = 0.001).

### Discussion

The Form Contingency condition received the highest affiliation score, confirming that repeating others’ behaviours within a specific frame of time serves to attribute affiliation. The Temporal Contingency condition does not differ from the No Contingency condition, which suggests that watching two individuals move at the same time is not enough to infer closeness between them. If anything, the slightly smaller mean in the Temporal Contingency condition might suggest that moving at the same time, but in different ways, may have a negative effect on perceived affiliation. We will return to this possibility in the discussion of our second study, which served to test the generalizability of our findings. A recent review by Cross, Turgeon and Atherton [24] suggests that the effect of mimicry depends on the pre-existing relationship between the interacting parties. Indeed, previous work with children has shown that coordinating behaviours leads to increased bonding only between members of different minimally created social groups [20], and similar results have also been found in adult samples with naturally created groups [24]. However, almost all mimicry-related research has been done with participants from modern, large-scale societies. Perhaps mimicry has developed as a mechanism for large-scale societies to coordinate the frequent interactions with members of other groups? In order to see whether mimicry has the same effect on people who still mostly interact with kin or other members of their ingroup, we took our study to Samburu, a traditional small-scale society in Northern Kenya (more details can be found in the Supporting Online Materials).

## Study II

### Method

#### Participants

In order to determine whether our results would hold across large- and small-scale populations, we tested young adults from a traditional pastoralist community in Kenya (Samburu). A total of fifty-eight students from Wamba Mixed High School between the ages of 15 and 22 (25 female, 33 male, M_*age*_ = 18.08, SD = 1.45) participated in our study. As in Spain, participation was fully voluntary and not part of the students’ course work. The study was approved by the ethical committee of University Jaume I and adhered to all applicable standards. In addition, an official license to carry out the relevant research was obtained from the Kenyan National Commission for Science, Technology, and Innovation (NACOSTI). Informed written consent was obtained from the participants’ guardian, and additional verbal consent was provided by each participant prior to commencing the study.

#### Materials

The same set of three videos described in Study I was used in Study II.

#### Design and procedure

In order to familiarize the Samburu participants with Likert ratings, they received a short training session prior to the actual study. After receiving an introduction to the general idea of Likert scales, the students were shown 15 PowerPoint slides displaying familiar images of animals, food and sports and were asked to rate them in terms of personal preference or similarity. By adding distractor questions about personal preferences we tried to avoid that participants would only focus on detecting similarities, which might have biased them when watching the videos. Students who were able to use the scale appropriately were then admitted to the actual test.

As with their Spanish peers, participants were randomly assigned to one of the three conditions: No Contingency (N = 18, M_*age*_ = 18.11, SD = 1.77), Temporal Contingency (N = 20, M_*age*_ = 18.15, SD = 1.34), and Form Contingency (N = 20, M_*age*_ = 18, SD = 1.29). An additional two students were tested in the No Contingency condition but excluded from analyses because both had marked two numbers on the Likert scale. The methods were identical to the ones described in Study I.

### Results

**Fig 2:**
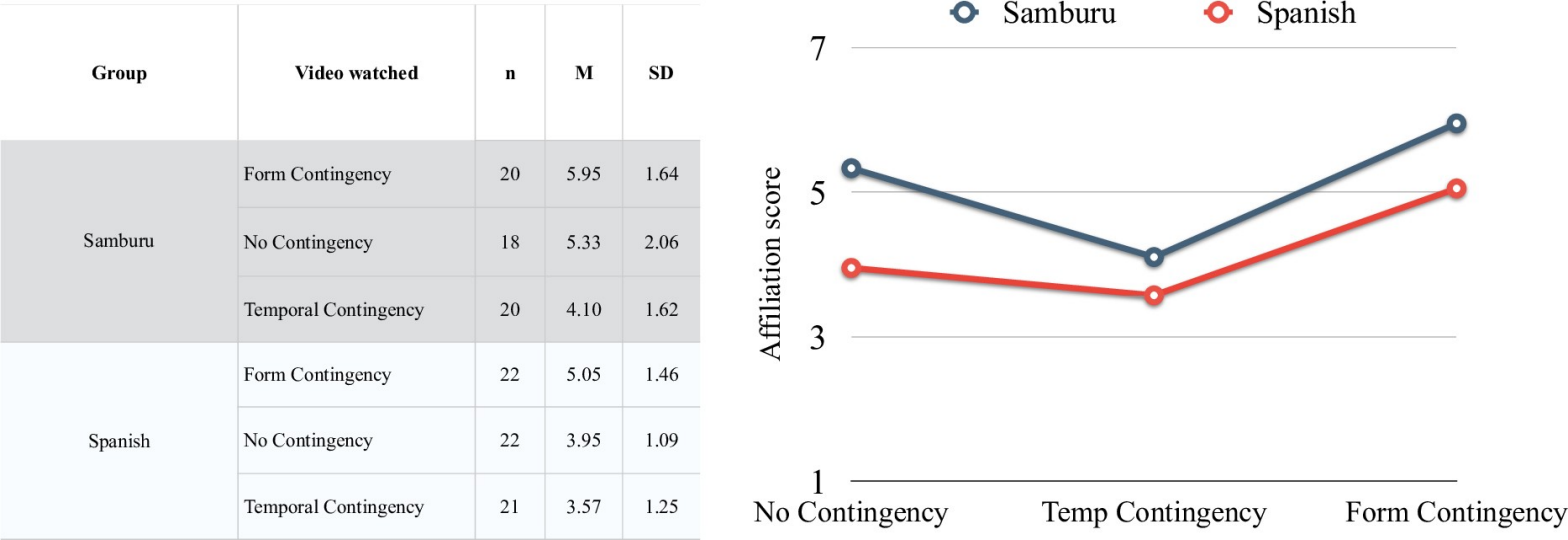
Means and standard deviations of the perceived affiliative status attributed to the actors as a function of the participant’s group and the video watched.

The results for the three conditions are shown in Fig 2 (upper graph in black). Visually, the Form Contingency condition again has the greatest effect on the affiliation scores, followed by the No Contingency and the Temporal Contingency conditions.

Unlike our Spanish sample, the Samburu did not significantly differentiate at the (α = 0.50 level between the No Contingency and the Form Contingency conditions (Tukey’s standardized d score = 0.62, p = 0.54), though qualitatively the pattern is the same. The failure to find a significant difference is not likely to result from low power for the test due to small sample size since the difference was not significant even at the α = 0.50 level. However, the Temporal Contingency condition differs significantly at the α = 0.05 level from the No Contingency condition (Tukey’s standardized d = 2.60, p = 0.011) and significantly at the α = 0.0001 level from the Form Contingency condition (Tukey’s standardized d = 4.51, p < 0.0001).

### Discussion

Compared to our Spanish sample, the Samburu assigned higher affiliation scores to the actors in the Form Contingency and No Contingency conditions, and relatively lower scores in the Temporal Contingency condition. However, the affiliation scores are not significantly different between the Form Contingency and the No Contingency conditions. While these latter results might suggest that mimicry indeed plays a less important role for affiliation in small-scale societies, there is an alternative explanation. People from a different population are often perceived as being more similar to each other than individuals from one’s own cultural group [25]. Both actors in the video were Europeans, and although we did try to eliminate ostensive cues by using a dark filter, it is still possible that the Samburu perceived the actors as being members of a different population. If that is the case, the actors might have resembled each other more from the Samburu’s point of view to begin with, which would explain the higher affiliation scores in the No Contingency and Form Contingency conditions. In the Temporal Contingency condition, both actors were moving at the same time but in different ways, making the difference between them starker than in the remaining two conditions in which their movements were spaced across time. This would also support our notion that the pattern is qualitatively comparable with that of our Spanish sample, though with a less attenuated quantitative effect for Samburu.

## Overall results

In order to get a closer look at the intercultural differences, we compared the data from both groups for each of our three conditions.

The degree of affiliation attributed to the actors in the Form Contingency condition is significantly different at the α = 0.05 level between the two groups (Tukey’s standardized d = 2.54, p = 0.012), suggesting that Samburu perceived both actors as being more similar. However, unlike their Spanish peers, the Samburu did not differentiate between the Form Contingency and the No Contingency conditions, which might also show that the Samburu perceived the two actors to be more similar to start with.

The Temporal Contingency condition, on the other hand, revealed no significant difference at the α = 0.20 significance level between the two populations (Tukey’s standardized d = 1.16, p = 0.25). Both the Spanish and the Samburu students did not consider moving at the same time to be a marker of affiliation. Temporal Contingency alone thus seems to draw participants’ attention on the fact that the actors are moving in different ways, rather than focusing on the fact that they are moving at the same time.

## Overall discussion

In line with previous work, the results from our Spanish sample show that coordinating each other’s actions does have effects which can be linked to the formation or recognition of a common collective identity [26].

As we discussed above, none of our post-hoc decision results can be attributed to the effect of sample size on the power of statistical decision making. When the two actors were moving at the same time while performing different gestures, they were given only low affiliation scores, but when they displayed the same gestures with a slight 4 to 5 seconds’ delay, the affiliation scores went up. These effects are very similar to the ones reported for other situations in which people mimic each other [27, see 2 for a comprehensive review]. However, in most previous work, the gestures between the interacting individuals were coupled in both time and form, making it difficult to disentangle the individual roles of these contingencies for fostering affiliation. Among the few researchers who have looked at the timing and the similarity of actions individually, Catmur and Heyes [23] as well as Hove and Raisen [17] found that temporal contingency of movements between two partners led to an increase in prosocial behaviours and affiliation, regardless of whether the partner’s actions were the same as one’s own. In a similar vein, dancers who followed the same music tempo encoded and remembered more information about each other as compared to dancers that moved to a different beat, independent of whether the movements matched in form [28]. Here we further isolated the timing of the gestures from their similarity in form between subjects, and critically also removed any external marking of rhythm that participants were to follow and showed that observers will not attribute affiliation to two individuals based on simultaneous movement alone. In the Temporal Contingency condition, the actors’ behaviours were perfectly coordinated in terms of time, yet their form never matched. Previous research has shown that the onset of movement is a very powerful attention-getter [29]. Thus, when attention is maximally caught by the actors’ movements, the focus will be on two people displaying different gestures, reversing any affiliative effect the temporal contingency might have created. While the different affiliation scores might also be due to variations in the similarity of the individual actions, the higher affiliation scores found in the No Contingency condition confirm the previous line of thought: although the actors still perform different movements, these movements do not happen at the same time, rendering the contrast between them less stark. It therefore seems to be the contingency in form which has the strongest effect on affiliation; especially if the behaviour is being reproduced within a tightly set time window [16]. Another reason for our contingency in time to have negative effects on perceived affiliation is the interaction being devoid of any rhythm. Previous investigations that reported a positive effect of synchrony on affiliation had participants move to the same rhythm/tempo [18,19], and it was perhaps this common rhythm which connected them. Therefore, when we talk of interactional synchrony it is the shared regular oscillation of the body movements, either in-phase or anti-phase, that connects participants, rather than moving at the same time per se. Having removed rhythm as the scaffolding underlying and connecting actors’ motions, their simultaneous movements cease to have any positive effects on affiliation and instead evoke distance.

The results from our small-scale sample seem to point in the same direction. Like their Spanish peers, the Samburu also attributed significantly lower degrees of affiliation to the actors in the Temporal Contingency condition, and gave higher affiliation scores to the actors in the Form Contingency and the No Contingency conditions. However, the fact that there was no significant difference between the No Contingency and the Form Contingency conditions could mean that the Samburu did not rely on mimicry in order to assign affiliation. On the other hand, the smaller difference between the affiliation scores in the two conditions could also be due to the fact that the actors were both Europeans and could therefore have resembled each other more in the eyes of our African participants. All in all, our results suggest that movements are used to judge the degree of affiliation between two individuals in both large- and small-scale societies. While moving in different ways at the same time seems to increase the perceived distance between two individuals, movements which match in form seem to invoke closeness and perhaps serve as some type of shibboleth for creating affiliation. Movements which match in time, but not in form, seem to have the opposite effect. The fact that the affiliation scores between the Form Contingency and the No Contingency conditions are attenuated in Samburu participants stresses the importance of designing universally valid methods for studying the affiliative effects of mimicking behaviours, and test them across a wide variety of populations.

## Supporting information

S1 Table No contingency. (PDF)

S2 Table Temporal contingency. (PDF)

S3 Table Form contingency. (PDF)

S1 Movie No contingency. (MOV)

S2 Movie Temporal contingency. (MOV)

S3 Movie Form contingency. (MOV)

S1 Data Raw data. (XLSX)

S1 File Samburu. (PDF)
