# Supplementary material for: Behavioural mimicry as an indicator of affiliation
Source: PLoS One. 2021 May 3;16(5):e0250105. doi: 10.1371/journal.pone.0250105 (PMC8092663; doi:10.1371/journal.pone.0250105)
Supplement: S1 Table — (PDF) [file pone.0250105.s001.pdf]

Table 1. Description of behaviours displayed by each actor in the No Contingency condition

NO CONTINGENCY

			Time action starts
**Starting posture**	Left	legs form a 90° angle, hands rest on knees	**0:00:00**
	Right	legs crossed, arms folded	**0:00:00**
**Action 1**	Left	scratches his head with his right hand	**0:00:05**
**Action 2**	Right	starts to yawn	**0:00:17**
**Action 3**	Left	stretches his legs and keeps them stretched, crosses his ankles (right one up); slightly moves his neck from right to left	**0:00:34**
**Action 4**	Right	legs forming a 90° angle, hands resting on knees	**0:00:47**
**Action 5**	Left	uncrosses his legs and keeps them aligned, touches his hair with his right hand	**0:01:07**
**Action 6**	Right	rubs his eyes as if he was tired (without exaggerating or changing his position)	**0:01:20**
**Action 7**	Left	brings in his legs and rests his right ankle on his left knee	**0:01:45**
**Action 8**	Right	keeps his position and crosses his arms	**0:02:04**
**Action 9**	Left	starts moving his legs rapidly, tapping the floor with his feet (while keeping his overall position)	**0:02:16**
**Action 10**	Right	keeps his position and visibly bites first his lower, and then his upper lip	**0:02:33**
**Action 11**	Left	again stretches his legs and crosses his ankles (right one up) hands resting on knees	**0:02:50**
**Action 12**	Right	keeps his overall position, straightens his back, slightly hunches his shoulders	**0:03:05**
**Action 13**	Left	keeps his position and folds his arms	**0:03:24**
**Action 14**	Right	keeps his position and crosses his ankles (right one up)	**0:03:38**
**Action 15**	Left	Legs form a 90° angle, hands resting on knees	**0:03:50**
**Action 16**	Right	starts to yawn	**0:04:02**
**Action 17**	Left	scratches his head with his right hand	**0:04:11**
**Action 18**	Right	keeps his position and slightly rubs his eyes	**0:04:26**
**Action 19**	Left	Keeps his overall position, starts tapping his fingers slightly on his knees, as if following a small rhythm	**0:04:42**
**Action 20**	Right	Stretches his arms, move his neck and cross his hands	**0:04:55**
**Action 21**	Left	keeps his position and scratches his head with his right hand	**0:05:08**
