# Supplementary material for: Behavioural mimicry as an indicator of affiliation
Source: PLoS One. 2021 May 3;16(5):e0250105. doi: 10.1371/journal.pone.0250105 (PMC8092663; doi:10.1371/journal.pone.0250105)
Supplement: S2 Table — (PDF) [file pone.0250105.s002.pdf]

Table 2. Description of behaviours displayed by each actor in the Temporal Contingency condition.

TEMPORAL CONTINGENCY

			**Time action starts**
**Starting posture**	Left	legs form a 90° angle, arms folded	**0:00:00**
	Right	legs crossed, arms folded	**0:00:00**
**Action 1**	Left	scratches his head with his right hand	**0:00:07**
	Right	keeps his overall position and begins to yawn	**0:00:07**
**Action 2**	Left	crosses his right leg and taps his toes	**0:00:26**
	Right	stretches his legs and keeps them stretched, crosses his ankles (right one up); slightly moves his neck from right to left	**0:00:26**
**Action 3**	Left	uncrosses his legs, touches his hair with his right hand	**0:00:46**
	Right	rubs his eyes as if he was tired (without exaggerating or changing his position)	**0:00:46**
**Action 4**	Left	aligns his legs and brings them into a 90° angle and hands rest of knees	**0:01:05**
	Right	keeps his leg position and folds his arms	**0:01:05**
**Action 5**	Left	starts moving his legs rapidly, tapping the floor with his feet (while keeps his overall position)	**0:01:25**
	Right	keeps his position and visibly bites first his lower, and then his upper lip	**0:01:25**
**Action 6**	Left	again crosses his ankles (right one up)	**0:01:46**
	Right	brings his legs into a 90° angle, straightens his back, slightly hunches his shoulders	**0:01:46**
**Action 7**	Left	keeps his leg position and folds his arms	**0:02:06**
	Right	keeps his overall position, unfolds his arms and hands rest on knees	**0:02:06**
**Action 8**	Left	keeps his overall position and begins to yawn	**0:02:26**
	Right	Scratches his head with his right hand	**0:02:26**
**Action 9**	Left	keeps his legs crossed and touches his hair with his right hand	**0:02:46**
	Right	keeps his overall position, starts tapping his fingers slightly on his knees, as if following a small rhythm	**0:02:46**
**Action 10**	Left	keeps his overall position and unfolds his arms	**0:03:05**
	Right	scratches his head with his right hand	**0:03:05**
**Action 11**	Left	gently rubs his eyes while keeping his overall position	**0:03:26**
	Right	yawns while keeping his overall position	**0:03:26**
**Action 12**	Left	keeps his overall position and starts moving his fingers	**0:03:45**
	Right	with his legs stretched out, crosses his right ankle over his left one	**0:03:45**
**Action 13**	Left	keeps his overall position and scratches his head with his right hand	**0:04:06**
	Right	keeps his overall position and aligns his outstretched legs	**0:04:06**
**Action 14**	Left	keeps his overall position and folds his arms	**0:04:25**
	Right	keeps his position and starts moving his right foot in circles	**0:04:25**
**Action 15**	Left	keeps his position and moves his neck from right to left	**0:04:45**
	Right	keeps his position and stretches his arms towards the ceiling	**0:04:45**
**Action 16**	Left	keeps his overall position and unfolds his arms	**0:05:06**
	Right	keeps his overall position and raises his right leg onto his left one	**0:05:06**
