# Supplementary material for: Behavioural mimicry as an indicator of affiliation
Source: PLoS One. 2021 May 3;16(5):e0250105. doi: 10.1371/journal.pone.0250105 (PMC8092663; doi:10.1371/journal.pone.0250105)
Supplement: S3 Table — (PDF) [file pone.0250105.s003.pdf]

Table 3. Description of behaviours displayed by each actor in the Form Contingency condition.

FORM CONTINGENCY			
			**Time action starts**
**Starting posture**	Left	legs form a 90° angle, arms folded	**0:00:00**
	Right	legs form a 90° angle, arms folded	**0:00:00**
**Action 1**	Left	scratches his head with his right hand	**0:00:15**
	Right	scratches his head with his right hand (or *does the same*)	**0:00:19**
**Action 2**	Right	unfolds his arms and rests his hands on his knees	**0:00:35**
	Left	unfolds his arms and rests his hands on his knees	**0:00:39**
**Action 3**	Left	crosses his right leg and taps his toes	**0:00:54**
	Right	crosses his right leg and taps his toes	**0:00:59**
**Action 4**	Right	keeps his legs crossed and touches his hair with his right hand	**0:01:15**
	Left	keeps his legs crossed and touches his hair with his right hand	**0:01:20**
**Action 5**	Left	stretches his legs and keeps them stretched, crosses his ankles (right one up); slightly moves his neck from right to left	**0:01:33**
	Right	stretches his legs and keeps them stretched, crosses his ankles (right one up); slightly moves his neck from right to left	**0:01:38**
**Action 6**	Right	rubs his eyes as if he was tired (without exaggerating or changing his position)	**0:01:53**
	Left	rubs his eyes as if he was tired (without exaggerating or changing his position)	**0:01:57**
**Action 7**	Left	aligns his legs and brings them into a 90° angle	**0:02:12**
	Right	aligns his legs and brings them into a 90° angle	**0:02:16**
**Action 8**	Right	keeps his leg position and folds his arms	**0:02:31**
	Left	keeps his leg position and folds his arms	**0:02:35**
**Action 9**	Left	starts moving his legs rapidly, tapping the floor with his feet (while keeping his overall position)	**0:02:49**
	Right	starts moving his legs rapidly, tapping the floor with his feet (while keeping his overall position)	**0:02:54**
**Action 10**	Right	keeps his position and visibly bites first his lower, and then his upper lip	**0:03:09**
	Left	keeps his position and visibly bites first his lower, and then his upper lip	**0:03:13**
**Action 11**	Left	again stretches his legs and crosses his ankles (right one up)	**0:03:28**
	Right	again stretches his legs and crosses his ankles (right one up)	**0:03:32**
**Action 12**	Right	keeps his overall position, straightens his back, slightly hunches his shoulders	**0:03:44**
	Left	keeps his overall position, straightens his back, slightly hunches his shoulders	**0:03:48**
**Action 13**	Left	keeps his leg position, unfolds his arms, and rests his hands on his knees	**0:04:06**
	Right	keeps his leg position, unfolds his arms, and rests his hands on his knees	**0:04:10**
**Action 14**	Right	keeps his overall position, starts tapping his fingers slightly on his knees, as if following a small rhythm	**0:04:25**
	Left	keeps his overall position, starts tapping his fingers slightly on his knees, as if following a small rhythm	**0:04:29**
**Action 15**	Left	brings his legs into a 90° angle while keeping his overall position	**0:04:45**
	Right	brings his legs into a 90° angle while keeping his overall position	**0:04:50**
**Action 16**	Right	keeps his overall position and begins to yawn	**0:05:02**
	Left	keeps his overall position and begins to yawn	**0:05:05**
