# Supplementary material for: Behavioural mimicry as an indicator of affiliation
Source: PLoS One. 2021 May 3;16(5):e0250105. doi: 10.1371/journal.pone.0250105 (PMC8092663; doi:10.1371/journal.pone.0250105)
Supplement: S1 File — (PDF) [file pone.0250105.s008.pdf]

Samburu

The Samburu are livestock herders living in the sparsely populated, arid plains of northern Kenya, and currently number around 300,000. They speak Samburu, a Nilotic language closely related to Maa. Unlike other ethnic groups in Kenya, the Samburu have largely upheld their cultural traditions and embraced Westernization to a much lesser degree. Economic activities are mainly organized on the household level (Holtzmann, 1996), and interactions with unrelated strangers are infrequent and typically limited to visits to smaller towns. Although most Samburu today own mobile phones, communication is often restricted by lack of network or power for charging, and media access through TV or internet is often restricted to urban centres.

Samburu social organization is largely based on age, with leaders being significantly older than the majority of the adult population (Spencer, 2013). Gender is an equally strong factor for determining social status, with males being the undisputed heads of the family. Children are highly valued, and families typically have five or more. Infants are treated leniently until they are about 3 years old, but as they grow older, the amount of obedience required increases. Most children start helping around the household when they are about four years old. Girls take care of younger siblings and help to fetch water and firewood, while boys typically look after the family's livestock. Small children are usually part of multi-age playgroups, and games involve both collaborative and competitive activities. Pretend play includes the typical mimicking of adult activities and animals; and imitating the movements of bulls, who play a major role in Samburu culture, is a central element of Samburu dance. Rituals also involve synchronizing with others, although both songs and dances often have a competitive component, in which participants try to outperform each other rather than matching their partners' moves in time and form (Spencer, 1985).

Holtzman, J. D. (1996). *Transformations in Samburu domestic economy: The reconstitution of age and gender-based processes of production and resource allocation among a Kenyan pastoral people* (Doctoral dissertation).

Spencer, P. (2013). *The Samburu: a study of gerontocracy in a nomadic tribe* (Vol. 26). Routledge.

Spencer, Paul (1985) 'Dance as Antithesis in the Samburu Discourse.' In: Spencer, P., (ed.), *Society and the Dance.* Cambridge University Press, pp. 140-164.
